# Reduced IL-33 plasma levels in aplastic anemia

**DOI:** 10.1186/s12935-015-0270-5

**Published:** 2015-12-16

**Authors:** Ming Sun, Hai-feng Ma, Ye-yun Che, Xin Cui

**Affiliations:** Department of Hematology, Linzi District People’s Hospital, Zibo, 255400 China; Department of Internal Medicine, Shandong Provincial Hospital Affiliated to Shandong University, Jinan, 250021 China

**Keywords:** Aplastic anemia, Interleukin 33, Interleukin 17, sST2, Cytokines

## Abstract

**Background:**

In this study, we aim to evaluate the balance of interleukin (IL)-33 and its soluble receptor sST2 in patients with aplastic anemia (AA).

**Methods:**

Plasma IL-33, IL-17 and sST2 levels were measured in patients with active AA (n = 31), AA in remission (n = 29) and in healthy subjects (n = 30), using enzyme linked immunosorbent assays (ELISAs).

**Results:**

The results showed that sST2 and IL-17 levels were significantly elevated in patients with active AA when compared to control subjects, but IL-33 levels were significantly lower in AA patients, which resulted in elevated sST2/IL-33 ratios in patients with active disease. During remission stages, the levels of these cytokines were comparable to those of healthy controls.

**Conclusions:**

The elevated levels of sST2/IL-33 in the plasma during active stages of the disease suggest a possible role in the pathogenesis and course of AA.

## Background

Acquired aplastic anemia (AA) is primarily considered an immune-mediated bone marrow failure syndrome, which differs from the other conditions associated with inherited mechanisms [1]. Environmental exposure to, for example, drugs, viruses, and chemical and physical toxins, is thought to trigger an aberrant immune response in some patients, but most cases are classified as idiopathic immune-mediated AA. Aberrant immunity, including abnormal immune cells and molecules, contributes to the development of acquired AA [2, 3]. It has become evident that T helper 1 (Th1) and Th2 cells have pathogenic importance in AA; activated antigen-presenting cells (APCs), particularly dendritic cells (DCs), may promote polarization to Th1 cells and activate cytotoxic T lymphocytes (CTLs). A variety of immune molecules, including interferon-γ (IFN-γ), tumor necrosis factor-α (TNF-α) and interleukins (ILs) produced by DCs, T lymphocytes and stromal cells, comprise a cytokine network for the destruction of hematopoietic stem cells [3–6].

Interleukin (IL)-33 (or IL-1F11) is the most recently discovered member of the IL-1 cytokine family, which was identified as a ligand for the T1/ST2 receptor (ST2; also called ST2 L, IL-33R, or IL-1RL1) [7]. Like IL-1, IL-33 appears to act as a dual function cytokine with both nuclear and extracellular effects [8]. The extracellular effects of IL-33 are mediated by binding to ST2 with subsequent recruitment of IL-1R accessory protein (IL-1RAcP), leading to the activation of NF-kB and MAPK pathways and signaling similar to IL-1. Like other members of the IL-1 receptor family, ST2 also exists in a soluble form (sST2), generated by alternative mRNA splicing, which acts as a decoy receptor to inhibit IL-33 signaling [7]. Over the last several years, several studies have suggested that IL-33 and sST2 are involved in immune diseases such as systemic lupus erythematosus and atopic dermatitis [8–10].

IL-17 can be produced by Th17 cells, as well as some other immune cells such as mast cells, neutrophils and macros. It is associated with autoimmune disease, infection disease and is treated as a therapeutic target in recent years [11–13]. However, data’s come from different research groups were controversial about the expression of IL-17 between AA patients and normal controls [14–16]. Here we further investigate the expression of IL-17, IL-33 and sST2 in patients with acquired AA.

In the present study, we hypothesized that the imbalance of IL-33 and sST2 may play an important role in acquired AA. Expression levels of IL-17, IL-33 and sST2 were measured in the serum of 31 newly diagnosed patients with active AA, 29 AA patients in remission and 30 healthy volunteers to investigate the possible role that IL-33 and sST2 play in AA.

## Methods

### Patients and controls

Thirty-one newly diagnosed AA patients (19 females and 11 males; age range, 16–61 years; median age, 27 years) were enrolled in this study. None of these patients had been transfused or treated by immunosuppressive therapy prior to sampling. Out of the 31 patients, 10 patients were diagnosed with severe AA (SAA) and 21 patients with moderate AA (MAA) according to the criteria described by Camitta et al. [17]. Marrow cytogenetics were normal in all cases, and Fanconi anemia was excluded in children and adolescents based on family history and the presence of typical physical characteristics. Patients complicated with diabetes, hypertension, cardiovascular diseases, pregnancy, active infection, or connective tissue diseases, such as systemic lupus erythematosus, were excluded. Twenty-nine AA patients (15 females and 14 males, age range 15–54 years, median 38 years) were in remission. All of the patients met the diagnostic criteria of AA by bone marrow biopsy and peripheral blood counts, as recommended by the International Study of Aplastic Anemia and Agranulocytosis [18]. Thirty healthy controls were included (18 females and 12 males; age range, 15–62 years; median, 34 years). Enrollment took place between July 2012 and January 2014 in the Linzi District People’s Hospital and Shandong Provincial Hospital Affiliated with Shandong University. Our research was approved by the Medical Ethical Committee of the Linzi District People’s Hospital and Shandong Provincial Hospital Affiliated with Shandong University. An informed consent document was obtained from each participant.

### Plasma preparation

Peripheral blood was collected into heparin-anticoagulant vacutainer tubes. Plasma was obtained from all subjects by centrifugation and stored at −80 °C for determination of cytokines. Mononuclear cells were isolated from heparinized blood by gradient centrifugation on Ficoll-Paque (Pharmacia Diagnostics, Uppsala, Sweden).

### IL-33, IL-17 and sST2 enzyme-linked immunosorbent assay (ELISA)

Plasma IL-33, IL-17 and sST2 were measured using commercial enzyme-linked immunosorbent assay (ELISA) kits according to the manufacturer (eBioscience and R&D, respectively) instructions. The lower detection limits of the kits for IL-33, IL-17 and sST2 were 7.8, 0.5 and 31.3 pg/ml, respectively. All samples were repeated three times.

### Statistical analysis

Data were expressed as the mean ± SD. Statistical significance was determined by one-way ANOVA using SPSS Windows version 13.0. A probability value *P* < 0.05 was considered statistically significant.

## Results

### Plasma concentrations of IL-33 and sST2 in ITP and controls

**Fig. 1:**
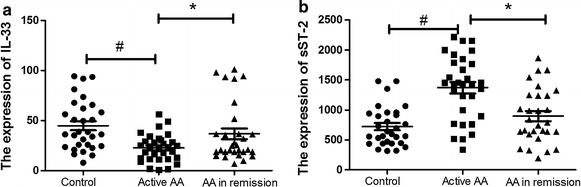
Plasma expression of IL-33 and sST2 in AA and controls. Plasma concentrations of IL-33 in AA patients with active disease (22.7 ± 13.7 pg/ml) were found to be significantly lower than those of healthy controls (45.8 ± 24.6 pg/ml, *P* < 0.01) and AA patients in remission (36.8 ± 29.5 pg/ml, *P* < 0.01) (**a**). Plasma sST2 levels of AA patients with active disease (1370.1 ± 530.3 pg/ml) were significantly higher compared with healthy controls (903.7 ± 329.3 pg/ml, *P* < 0.01) and patients in remission (1331.8 ± 454.3 pg/ml, *P* < 0.01) (**b**)

As shown in Fig. 1a, plasma concentrations of IL-33 in AA patients with active disease were found to be significantly lower than those of healthy controls (22.7 ± 13.7 vs. 45.8 ± 24.6 pg/ml, *P* < 0.01). Plasma sST2 levels of AA patients with active disease were significantly higher compared with healthy controls (1370.1 ± 530.3 vs. 903.7 ± 329.3 pg/ml, *P* < 0.01; Fig. 1b). Active AA patients had significantly lower IL-33 and significantly higher sST2 plasma levels than AA patients in remission (22.6 ± 13.4 vs. 36.8 ± 29.5 pg/ml, *P* < 0.01 and 1370.1 ± 530.3 vs. 1331.8 ± 454.3 pg/ml, *P* < 0.01, respectively). However, no significant difference was found in plasma levels of IL-33 and sST2 between AA patients in remission and healthy controls.

### The ratio of sST2/IL-33 in normal control patients, patients with active disease and patients in remission

**Fig. 2:**
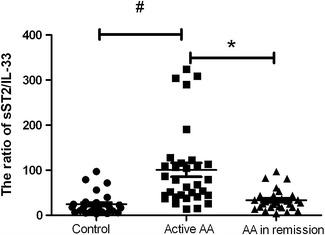
The ratio of sST2/IL-33 in normal control and AA patients. ^#^ *P* < 0.05, active AA vs controls. *P < 0.05, active AA vs AA in remission

The IL-33 plasma levels of AA patients with active disease were significantly lower than the normal controls, and those patients had significant higher sST2 plasma levels than the control group. The ratio of sST2/IL-33 in patients with active disease was significantly higher the ratio in the normal and remission groups (*P* < 0.01), but there was no significant difference in the sST2/IL-33 ratio between patients in remission and the normal group (Fig. 2).

### Plasma concentration of IL-17 in ITP and controls

**Fig. 3:**
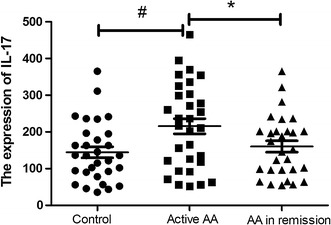
Plasma expression of IL-17 in AA and controls. ^#^ *P* < 0.05, active AA vs controls. *P < 0.05, active AA vs AA in remission

Plasma IL-17 levels were significantly higher in AA patients with active disease than in healthy controls (215.5 ± 115.5 vs. 144.3 ± 81.6 pg/ml, P < 0.01). Significantly lower IL-17 plasma levels were found in AA patients in remission compared with active AA patients (160.0 ± 83.1 vs. 215.5 ± 115.5 pg/ml, P < 0.01). However, no significant difference in IL-17 plasma level was found between AA patients in remission and healthy controls (Fig. 3). There was no correlation between IL-17, IL-33 or sST2 level or white blood cell, hemoglobin, and platelet counts in the individuals examined in the present study.

### The correlation between IL-17 with IL-33 or sST2 in active AA patients

**Fig. 4:**
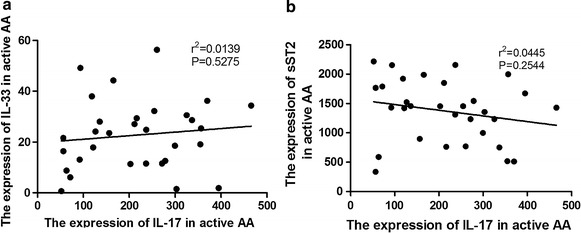
The correlation between IL-17 and IL-33 or sST2 in active AA patients. There was no correlation between IL-17 and IL-33 (r^2^ = −0.0139, P = 0.5275; N = 31; **a**) or sST2 (r^2^ = −0.0445, P = 0.2544; N = 31; **b**) expression in active AA patients

Spearman correlation analysis was used to evaluate whether IL-17 expression was correlated with IL-33 or sST2. As shown in Fig. 4, there was no correlation between IL-17 and IL-33 (r^2^ = −0.0139, P = 0.5275; N = 31; Fig. 4a) expression or between IL-17 and sST2 (r^2^ = −0.0445, P = 0.2544; N = 31; Fig. 4b) expression in active AA patients.

## Discussion

Patients with active AA disease demonstrated decreased levels of IL-33 expression, while the levels of sST2 in patients were comparable upregulate to the levels of healthy controls. And IL-17 was higher in AA patients with active disease. There was no correlation between IL-17 and IL-33 expression.

AA is a rare, potentially life-threatening failure of hematopoiesis characterized by pancytopenia and bone-marrow aplasia; AA was originally thought to result from a direct toxic effect on hematopoietic stem cells that led to a decrease in their numbers. Increased monocyte-derived DCs (DC1) shifted from the stable form to an active one and promoted Th0 cells to polarize into Th1 cells. The alterations in Th1 lymphocyte function and cytokines have been intensively investigated as surrogates of AA and as possible candidates of an index for diagnosis and prognosis [18, 19].

IL-33 is a pro-inflammatory cytokine belonging to the IL-1 family, though its biology presents some differences from that of IL-1b. The IL-33 pathway plays a major role in immune and inflammatory responses by inducing Th2 immune responses [20, 21]. Aberrant IL-33 expression in patients with autoimmune disorders suggests the participation of this inflammatory cytokine in the initiation and progression of diseases including asthma [22], systemic lupus erythematosus and inflammatory arthritis [23]. Because abnormal expression of IL-33 may lead to the autoimmune disease mentioned above, targeting IL-33 is likely to provide potential opportunities for the treatment of this disorder. The observed reduction in IL-33 levels in AA patients was more obvious than in AA patients in remission, thereby implying that reduced IL-33 expression may lead to more intensive T cell accumulation. Therefore, up-regulating expression of IL-33 in AA patients may also represent a therapeutic approach against AA.

Suppression of tumorigenicity 2 (ST2) is a member of the IL-1 receptor (IL-1R) family that plays a major role in immune and inflammatory responses. Alternative promoter activation and splicing produces both a membrane-bound protein (ST2 L) and a soluble form (sST2). The transmembrane form of ST2 is selectively expressed on Th2- but not Th1-type T cells, and binding of its ligand, IL-33, induces Th2 immune responses. In contrast, the soluble form of ST2 acts as a decoy receptor by sequestering IL-33 [24]. The IL-33/ST2 pathway has important immunomodulatory effects [20]. Tajima S found that ST2 protein increased in the serum, reflecting severity in the inflammatory process and Th2 immune response in the IPF lung [25]. Martínez et al. reported that sST2 is increased in aorta from obese rats, and in addition is inducing vascular fibrosis, suggesting that sST2 could participate in the remodeling observed in obesity [26]. In our present study, AA patients had a relatively increased sST2 level compared to controls. sST2 may act not only as a decoy receptor by binding IL-33 and preventing ST2L signaling, but also exerts its direct effects modulating immune responses in AA. Further understanding of the molecular mechanisms by which sST2 regulates aberrant immunity may lead to novel targeted therapies for AA.

IL-17, which can be secreted by Th17 cells, was treated as a therapeutic target in some autoimmune disease [27], which promote secretion of multiple inflammation factors like TNF-α, IL-1, IL-6 and IL-8. IL-17 can also inhibit proliferation of human hematopoietic progenitor [28, 29]. Our study confirmed IL-17 expression in plasma was upregulated in AA, which in accord with earlier research [16]. Recent, evidence supports the idea of IL-33 as an inductor of immune response by influencing different regulatory cell populations expressing its receptor. Additionally, it has been shown in experimental autoimmune encephalomyelitis that IL-33 is capable of diminishing the secretion of inflammatory cytokines such as IFN-γ (Th1) and IL-17 (Th17) [30]. The decreased levels of IL-33 may induce upregulation of IL-17, then regulating the balance of IL-33 and sST2 in AA patients may also recover IL-17 expression.

Our results confirmed a reciprocal pattern in expression of IL-33 and sST2 in AA; the levels of IL-33 were significantly decreased in active AA patients than in controls and patients in remission, while sST2 levels were not significantly changed in AA patients. The ratio of sST2/IL-33 in patients with active disease was significantly increased when compared with that of the normal groups and AA patients in remission. Regulating the balance of IL-33 and sST2 in AA patients may also be a therapeutic approach against AA, although further studies are warranted.
